# Opium, in Fever, in Large Doses

**Published:** 1845-10-01

**Authors:** 


### EDITORIAL, MEDICAL INTELLIGENCE, &c.

*Opium in Fever, in large doses.*—The Missouri Medical and Surgical Journal for August last, contains an article on the above subject, by A. G. Henry, M. D., of Pekin, Illinois. Dr. Henry advocates the administration of opium in doses of four or five grains in all fevers, after emetics, bleeding and cathartics, provided the symptoms have indicated the latter remedies. He states that he has pursued this course for the last 8 years, in the remittent, typhoid, and congestive fevers, which have successively prevailed, more or less, in his locality. He usually combines with the opiates calomel in large doses. Even when complicated with cerebral affection, he does not hesitate to use it freely after free depletion. He states that in nineteen out of twenty cases, he administers it so soon as the stomach becomes settled after the operation of an emetic given at the onset of the disease. When gastric irritation exists, rendering an emetic inadmissible, he gives it without any delay.

The Doctor is evidently a practical man, and we are disposed to attach considerable value to his experience. We should have been better satisfied, however, if he had given his observations with more precision. We do not perceive that his inductions are based on facts carefully recorded and collated. The longer we reflect and observe, the more we are convinced that unrecorded experience is extremely delusive. Nothing is easier than to commit a rash generalization, and, afterward, [honestly enough,] see developed on every side, facts to confirm it. Hence, our science is proverbially prolific in false facts. The collection of sufficient data, and their rigorous analysis and comparison, before we commit ourselves to a new and important practical principle, will prevent a vast many errors which are difficult to be unlearned. We do not make these remarks with especial reference to the article by Dr. Henry, but because they have been incidentally suggested.

The Doctor adduces one consideration concerning his treatment of fevers, which applies to all therapeutical improvements. He states that the aggregate amount of his bills since he has pursued the opium plan is less than one half, in a given number of cases, of what it was previously! This is a consideration which, unfortunately, the physician can generally appreciate better than the patient. Persons generally attribute to the physician skill in proportion to the amount of medicine which he has given. It is not considered whether by a skilful tact the remedies have *hit the mark* at once, and, therefore, accomplished the end; but how much ammunition has been expended, and how long the attempt has been continued! It is, indeed, sometimes disheartening to witness the gratitude of the poor sufferer, who has been physicked until human nature could endure no more, and then by necessity left to the vis medicatrix, which, in spite of all, proves adequate to restoration; and the practical exemplification of this gratitude in the cheerful payment of a *long bill;* when, on the other hand, after the speedy issue of a judicious practice, the patient perhaps is dissatisfied that in so trifling a case he should have incurred the expense of any medical attendance, thinking that if he was so soon and easily restored, he could not have been very ill! The enlightened physician cannot compete with the ignoramus in the *pecuniary* avails of his services, provided both have an equal circle of friends. The latter will be *well to do*, while the former suffers the anxieties and mortifications of straightened means.—Yet it is a fact most honorable to the character of our profession, that few, if any of its members find in this consideration any motives for ignorance, or abate on this account their desire of improvement. That the profitable course which we have implied as the involuntary result of ignorance, is ever designedly pursued, is sometimes charged upon our profession, but we much doubt if the libel has even a single instance to support it.

